# Dysregulated connexin 43 in HER2-positive drug resistant breast cancer cells enhances proliferation and migration

**DOI:** 10.18632/oncotarget.22678

**Published:** 2017-11-25

**Authors:** Elizabeth S. Yeh, Christina J. Williams, Carly Bess Williams, Ingrid V. Bonilla, Nancy Klauber-DeMore, Stephanie L. Phillips

**Affiliations:** ^1^ Department of Cell and Molecular Pharmacology and Experimental Therapeutics, Medical University of South Carolina, Charleston, SC, USA; ^2^ Department of Surgery, Medical University of South Carolina, Charleston, SC, USA; ^3^ Department of Pediatrics, Division of Pediatric Hematology/Oncology, Medical University of South Carolina, Charleston, SC, USA

**Keywords:** connexin 43, gap junctions, HER2+ breast cancer, cell migration, resistance

## Abstract

Connexin 43 (Cx43) is a gap junction protein whose function in the development of breast cancer and in breast cancer progression remains unclear. Evidence suggests that Cx43 (*GJA1*) mRNA and protein expression is altered in breast tumors. However, reports indicate both increased and decreased Cx43 levels in human breast cancer samples. Studies also suggest that loss of Cx43 regulated gap junction intercellular communication is a common feature of breast malignancies that potentially correlates with histological stage. Further evidence suggests that Cx43 (*GJA1*) mRNA expression is negatively correlated with HER2 positivity but a relationship between Cx43 and HER2 in breast cancer is not well defined. Therefore, in this study, we sought to evaluate the relationship between Cx43 activity, HER2, and drug resistance. Using HER2+ breast cancer cell lines that are sensitive or resistant to HER2 inhibitor, we evaluated Cx43 gap junction function. We found that Cx43 gap junction activity is completely lost in drug resistant HER2-positive (HER2+) breast cancer cells, whereas Cx43 gap junction activity can be restored by Cx43 overexpression in drug sensitive HER2+ cells. Moreover, the dysregulation of Cx43 resulted in increased tumorigenic and migratory capacity of the HER2+ drug resistant breast cancer cells.

## INTRODUCTION

Connexins are the primary protein components of gap junctions, which are structures composed of aggregates of intercellular channels that facilitate direct cell-to-cell communication. More than 50 years ago, Loewenstein and colleagues first published that cancer cells lose their ability to communicate via gap junctions, highlighting the importance of intercellular communication for normal cell homeostasis [1]. Consequently, a potential benefit of targeting connexins in cancer is the ability to restore cell-to-cell communication, and possibly gap junction-mediated propagation of death signals, the so-called bystander effect. Combining this property with a drug cocktail has the potential to amplify the desired chemotherapeutic properties of cancer agents as well as reversing the inherent cancer promoting cellular properties of improperly functioning connexins.

Connexin 43 (Cx43) is arguably the most well studied of the connexin family members in breast cancer. Reports from studies examining human breast cancer tissue samples indicate that levels of Cx43 both increase and decrease with breast cancer stage [2–5]. In terms of activity, the current prevailing theory is that Cx43 gap junction intercellular communication (GJIC) is reduced in early stages of breast cancer as well as during the initial metastatic steps [6, 7]. Experimental studies suggest that reduced Cx43 expression levels or a diminished capacity for Cx43-dependent GJIC promotes breast cancer cell migration. [8–15]. It has also been suggested that Cx43 gap junctional intercellular communication between tumor cells and vasculature is potentially facilitative during later stages of metastasis involving extravasation and colonization in the secondary metastatic site [16–19]. Other areas of experimental investigation highlight non-canonical (i.e. non-gap junction related) functions for Cx43 and their importance in regulating cellular functions including cancer cell migration [20].

A major roadblock in furthering Cx43 research in breast cancer, as well as other human cancers, arises from the standard of using expression levels either at the genomic or protein level as a surrogate for Cx43 activity. While potentially useful, the idea that expression levels directly corresponds to activity levels may be inaccurate. Increased Cx43 levels do not necessarily equate with increased GJIC, particularly if the increase in Cx43 is coupled with cytoplasmic localization of the protein. Our current studies highlight this problem and serve to illustrate that there is a more complicated system of regulation that should be investigated in detail to appropriately facilitate Cx43-specific drug targeting and translation.

Previous evidence suggests that Cx43 expression levels are negatively correlated with HER2 expression [21, 22]. Additional experimental evidence indicates that HER2+ breast cancer cell lines have reduced Cx43 expression as compared to ER+ breast cancer cells [23, 24]. Somewhat paradoxically, the analysis presented here, suggests that high Cx43 expression levels correlates with reduced relapse free survival (RFS) in HER2-positive (HER2+) breast cancer. Since neither the former nor the latter analyses assessed Cx43 activity, we initiated an investigation to evaluate Cx43 protein activity and GJIC in HER2+ breast cancer cells. We posited that in HER2+ breast cancer cells that are responsive to HER2 inhibitors, GJIC could be restored by modulation of Cx43. However, in drug resistant cells the right opportunities or conditions are less favorable because additional mechanisms have arisen in drug resistant cells that further block Cx43 GJIC. To test this idea, we evaluated Cx43 function in HER2 inhibitor sensitive and resistant breast cancer cells and found that in fact, the drug resistant cells were clearly incompetent in their ability to propagate Cx43 GJIC. We put forth the conjecture that the more severe phenotype of Cx43 dysregulation in drug resistant cells is likely detrimental due to the cancer promoting capacity of losing GJIC, not necessarily directly from the loss of GJIC, but rather from the shift towards non-canonical functions for Cx43.

## RESULTS

### Different levels of Cx43 (GJAI1) mRNA but not protein are expressed in drug sensitive and drug resistant HER2+ breast cancer cells

**Figure 1:**
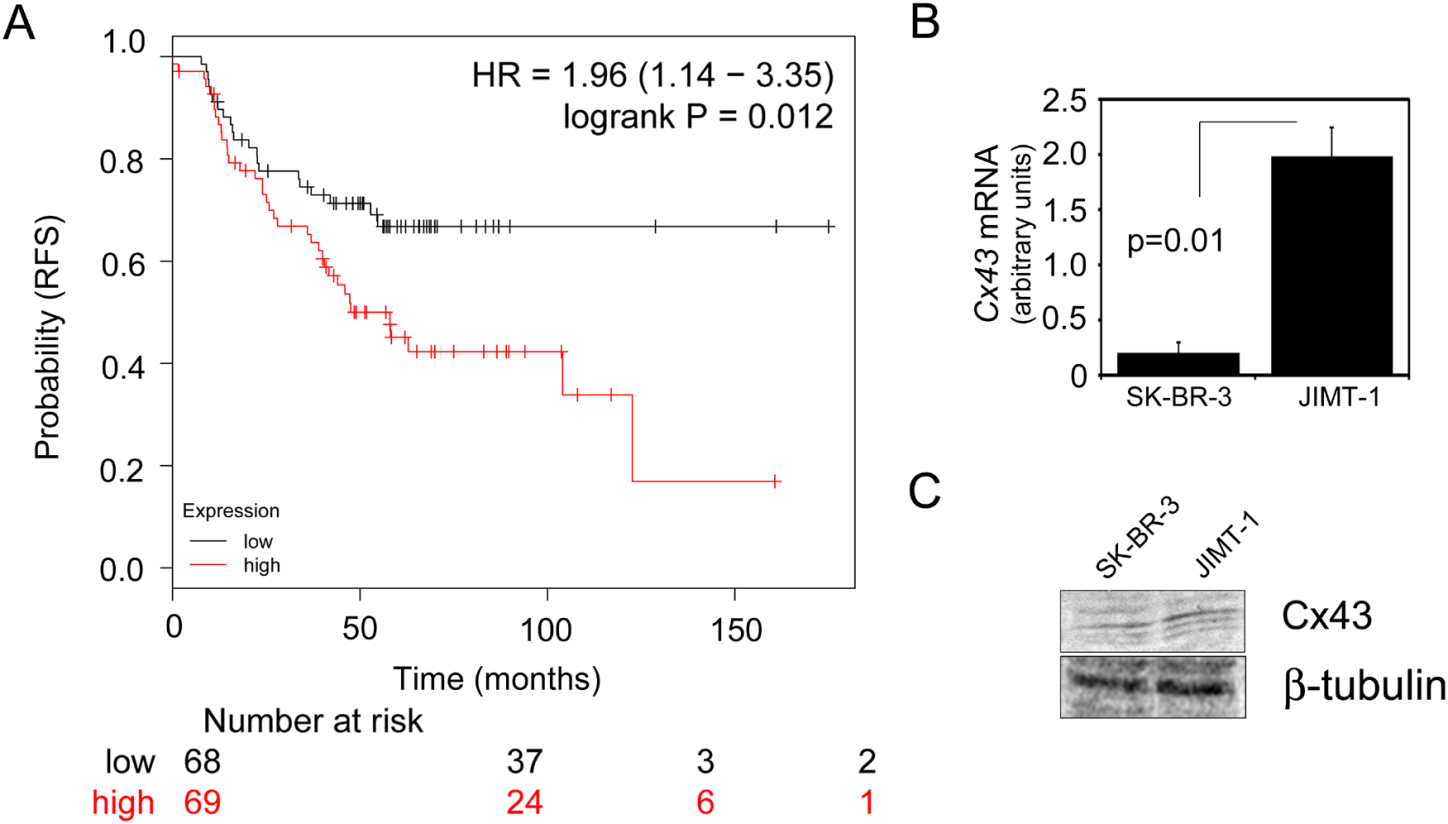
Cx43 (GJAI1) mRNA is elevated in JIMT-1 cells compared to SK-BR-3 cells but Cx43 protein is not **(A)** *GJA1* expression is associated with reduced relapse free survival (RFS) in HER2^+^/ErbB2 patients. Gene probe *GJA1* 201667_at was used for analysis with HER2^+^ status set to “positive” and ER status set to “negative” yielding n=137 patient samples with available clinical data containing the selected events. A total of n=68 patients were scored as “low” *GJA1* and n=69 were scored as “high” *GJA1.* Analysis tool automatically removed redundant samples and excluded any biased arrays. The probe expression range was classified as 73-16584 with a cutoff value of 2320 used for analysis. HR=1.96, logrank p-value=0.012. **(B)** Quantitative RealTime PCR assessment of Cx43 (*GJA1*) mRNA expression levels in SK-BR-3 compared with JIMT-1 breast cancer cells. JIMT-1 cells had ∼8-fold higher levels of *GJA1* mRNA in relation with SK-BR-3. *GJA1* levels were normalized to *GAPDH*. Student’s T-test indicated a p-value of p=0.01, n=3. **(C)** Western blot analysis of endogenous Cx43 protein levels in SK-BR-3 and JIMT-1 cells.

To evaluate the significance of Cx43 gene expression levels in HER2+ breast cancer, we used the Kaplan-meier plotter database tool (kmplot.com) to assess whether Cx43 (*GJA1*) gene expression correlates with relapse free survival (RFS) in HER2+ patients [25]. Using this tool, a gene probe for *GJA1* (201667_at) was used for analysis with HER2 status set to “positive” and ER status set to “negative” yielding n=137 patient samples with available clinical data containing the selected events. A total of n=68 patients were scored as “low” *GJA1* and n=69 were scored as “high” *GJA1.* The analysis tool automatically removed redundant samples and excluded any biased arrays. The probe expression range was classified by the Km plotter tool as 73-16584 with a cutoff value of 2320 used for analysis. Our analysis showed that *GJA1* expression correlates with reduced RFS (Figure 1A), which is somewhat inconsistent with previously published evidence suggesting that Cx43 expression levels are negatively correlated with HER2 expression [21, 22]. Since the expression analysis does not evaluate Cx43 protein levels or function, we asked whether the regulation of these aspects could differ between a HER2+ cell line that was sensitive to HER2 inhibitors compared to one that was resistant. Therefore, we first evaluated Cx43 (*GJA1*) gene expression by quantitative RealTime PCR in SK-BR-3 cells (drug sensitive) and JIMT-1 cells (drug resistant), two commonly used HER2+ cell models. Consistent with the gene expression analysis where higher expression of Cx43 correlated with poorer outcome (Figure 1A), *GJA1* levels in the drug resistant JIMT-1 cells were higher than the drug sensitive SK-BR-3 cells (Figure 1B). However, when we evaluated endogenous Cx43 protein expression and compared this between cell lines, there was no difference in Cx43 protein levels (Figure 1C and Supplementary Figure 1). These findings suggested to us that Cx43 has multiple nodes of regulation in breast cancer cells and evaluating gene expression is potentially not indicative of protein regulation or function.

### HER2 inhibitor resistant breast cancer cells are gap junction deficient

To determine if either of the HER2+ cell lines are Cx43 GJIC competent, we first evaluated baseline gap junction activity in each cell line using a parachute cell coupling assay. For this assay, 5000 “donor” cells were loaded with a cell permeable dye called Calcein AM. These cells were then dropped onto “acceptor” cells to evaluate dye transfer from the donor cells to the acceptor cells. Interestingly, neither the SKBR3 (sensitive) nor the JIMT-1 (resistant) cell line had any baseline gap junction activity since neither had increased dye transfer above the initial 5000 cells that were loaded with Calcein AM as donor cells (Supplementary Figure 2). This observation is consistent with the idea the HER2+ breast cancer cells reduce their Cx43 protein expression levels resulting in impaired GJIC.

**Figure 2:**
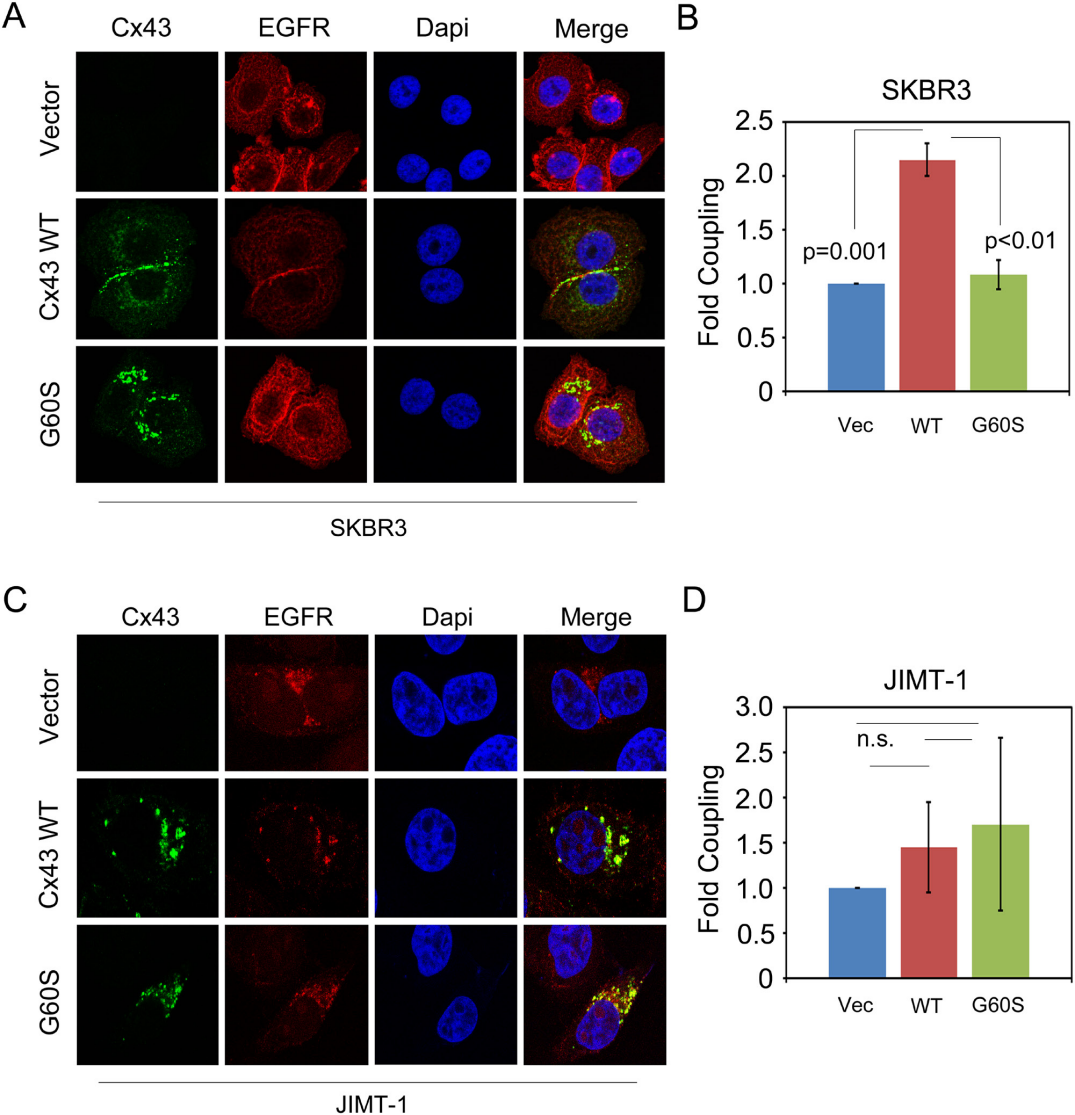
The ability to restore Cx43 GJIC in JIMT-1 cells is compromised **(A)** SK-BR-3 cells expressing either a vector control, Cx43, or Cx43 G60S were stained with anti-Cx43 antibody to detect Cx43 and anti-EGFR antibody to demarcate cells. Nuclei were stained with Hoechst dye. **(B)** Quantitation of parachute assay comparing SK-BR-3 cells expressing either a vector control, Cx43, or Cx43 G60S. **(C)** JIMT-1 cells expressing either a vector control, Cx43, or Cx43 G60S were stained with anti-Cx43 antibody to detect Cx43 and anti-EGFR antibody to demarcate cells. Nuclei were stained with Hoechst dye. **(D)** Quantitation of parachute assay comparing JIMT-1 cells expressing either a vector control, Cx43, or Cx43 G60S.

We next overexpressed Cx43 in each cell line by stable retroviral transduction because prior reports indicate that overexpression is a method that can drive the restoration of GJIC in breast cancer cell lines [19, 26–28]. We also stably transduced cells with a mutant of Cx43 that contains a Glycine to Serine amino acid substitution, G60S. This mutation has previously been shown to render Cx43 gap junction communication deficient [29]. To confirm expression, we first assessed these cell lines by immunofluorescence, which revealed a dramatic difference in localization when evaluating Cx43 in the drug sensitive (SK-BR-3) compared to the drug resistant (JIMT-1) cell line. In the drug sensitive SK-BR-3 cells, Cx43 appeared to form large plaques that localized at the plasma membrane between two neighboring cells, indicative of gap junctions (Figure 2A). Conversely, Cx43 in drug resistant JIMT-1 cells was always localized in punctate intracellular structures (Figure 2C). The Cx43 G60S mutant localized in punctate intracellular structures in both the SK-BR-3 and the JIMT-1 cells suggesting loss of GJIC as expected (Figure 2A and 2C). EGFR was used in these experiments to demarcate cells. Though not a measure of activity, this observation suggests that SK-BR-3 cells maintain the capacity to drive Cx43 gap junction plaque formation whereas JIMT-1 cells appear to have lost this capacity, despite both cell lines exhibiting low endogenous expression of Cx43 (Figure 2A and 2C, top panels, Figure 1C, and Supplementary Figure 1).

To directly test the ability of each of the Cx43 expressing cell lines to propagate GJIC, we performed cell coupling analysis. Our results indicate that overexpression of Cx43 induced coupling in SK-BR-3 cells, indicating that the mechanisms allowing Cx43 to propagate GJIC is intact in these cells (Figure 2B). Expression of Cx43 G60S in SK-BR-3 cells did not induce GJIC (Figure 2B). However, when we evaluated the same properties in JIMT-1 cells, Cx43 overexpression did not induce coupling, nor did the Cx43 G60S mutant, suggesting that Cx43 GJIC is impaired in JIMT-1 cells (Figure 2D). Taken together, these findings indicate that although Cx43 GJIC is impaired during breast cancer malignancy in HER2+ cells that remain sensitive to HER2 inhibitor, likely due to downregulation of total Cx43 levels, GJIC is rescuable by Cx43 overexpression. Interestingly, the gap junction activity of Cx43 protein appears to be further compromised during the acquisition or establishment of resistance in a manner that prevents exogenous expression of Cx43 from rescuing Cx43 GJIC in HER2 inhibitor resistant cells.

### Cx43 expression in HER2 inhibitor resistant cells leads to increased capacity for proliferation

**Figure 3:**
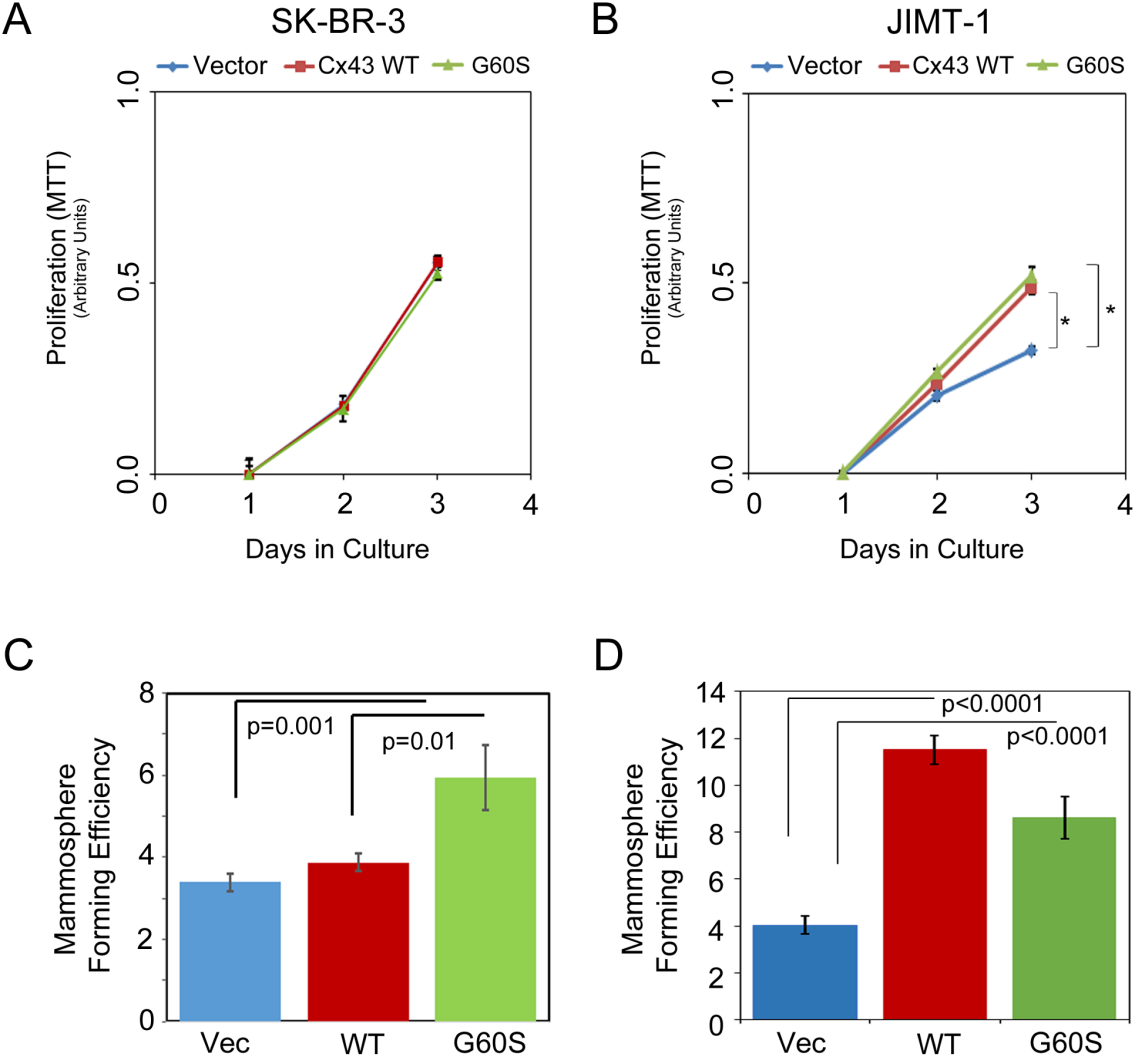
Cell proliferation and mammosphere formation in SK-BR-3 and JIMT-1 cells expressing Cx43 **(A)** MTT analysis of SK-BR-3 expressing a vector control, Cx43, or Cx43 G60S. Proliferation was assessed over the course of 3 days. **(B)** MTT analysis of JIMT-1 expressing a vector, Cx43, or Cx43 G60S. Proliferation was assessed over the course of 3 days. **(C)** SK-BR-3 expressing vector control, Cx43, or Cx43 G60S were placed under conditions for assessing mammosphere formation. Mammosphere forming units were quantitated and student’s T-test was performed to determine p-values, p=0.01 as indicated, n=32 sample wells per experiment. **(D)** JIMT-1 expressing vector control, Cx43, or Cx43 G60S were placed under conditions for assessing mammosphere formation. Mammosphere forming units were quantitated and student’s T-test was performed to determine p-values, p<0.001 as indicated, n=32 sample wells per experiment.

Since prior reports indicate that Cx43 regulates cellular functions including proliferation through non-canonical signaling [30–32], we next wanted to determine whether the differences we observed in Cx43 protein regulation in the HER2 inhibitor sensitive and resistant cell lines affect cellular proliferation and viability. First, we evaluated proliferation by MTT assay in the SK-BR-3 and JIMT-1 control, Cx43, or Cx43 G60S overexpressing cells. We found that Cx43 overexpression increased proliferation in the JIMT-1 (resistant) cells (Figure 3B) but not the SK-BR-3 (sensitive) cells (Figure 3A). The effect of the Cx43 G60S mutant in each cell line mirrored the phenotype of the wild type (WT) Cx43 (Figure 3A and 3B). Following, we evaluated viability under serum limiting conditions in each cell type. Each cell line was plated in equal number and then subjected to serum deprivation the following day. Total cell number was evaluated prior to serum deprivation (0hr) and at 48hr post-serum withdrawal. We found that JIMT-1 cells expressing Cx43 survived and multiplied ∼4-fold during this period whereas cell proliferation remained static under these conditions in the SK-BR-3-Cx43 cell population (Supplementary Figure 3). We noted that cell number did not decrease in any of the populations under serum limiting conditions, suggesting no inherent loss in viability. Therefore, these findings indicated to us that the dysregulation of Cx43 GJIC activity in the HER2 inhibitor resistant JIMT-1 cells results in increased proliferation.

### Non-junctional Cx43 is a feature of HER2 inhibitor resistant breast cancer cells that drives tumorigenesis

**Figure 4:**
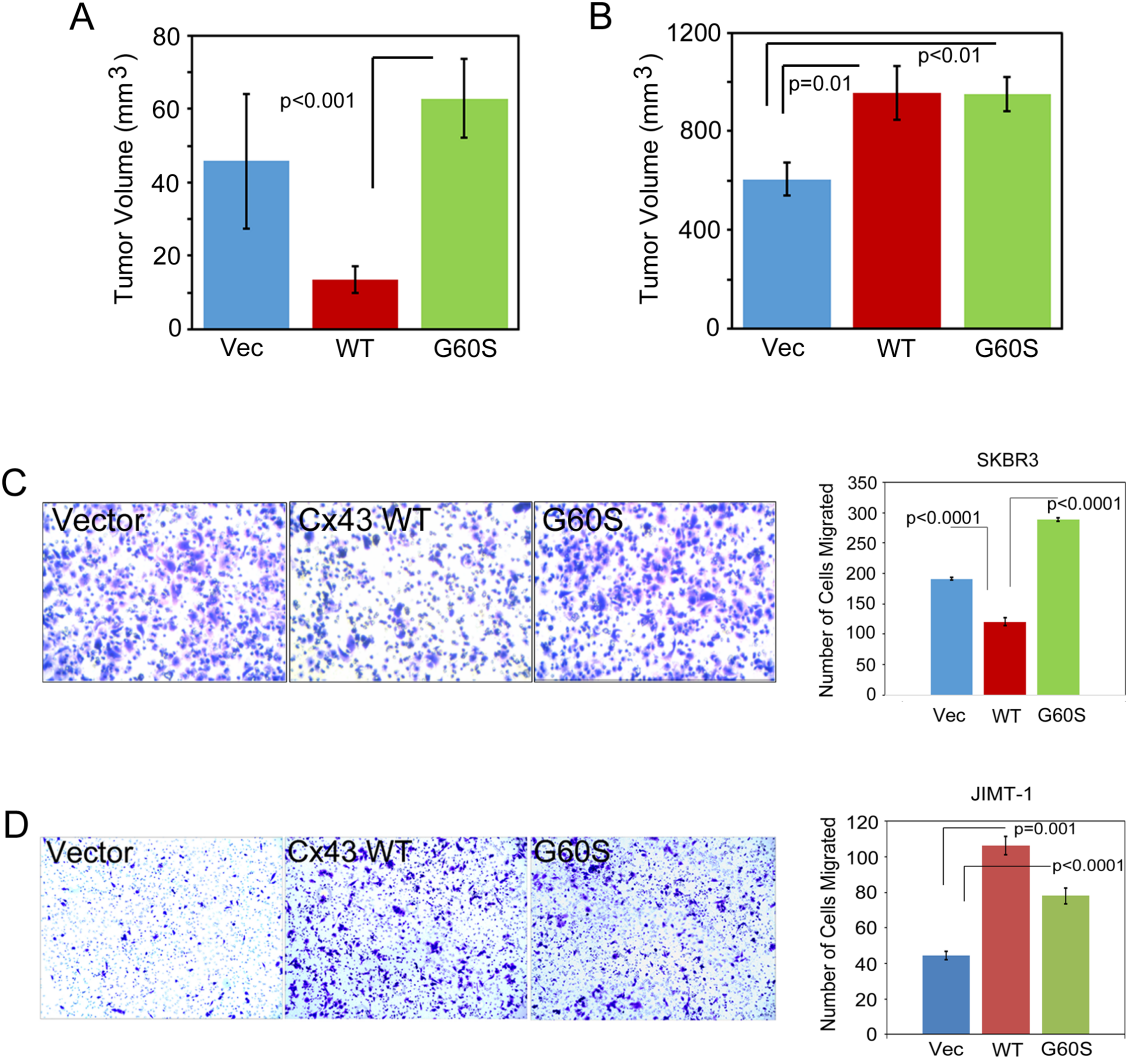
Expression of Cx43 in JIMT-1 cells promotes tumor growth and cell migration **(A)** SK-BR-3 expressing vector control, Cx43, or Cx43 G60S were injected into the mammary fat pad of immunocompromised mice to assess mammary tumor xenograft formation and growth. Tumor volume analysis on day 32 post-injection is presented. Student’s T-test was performed to determine p-values, p<0.001 as indicated, n=10 animals per group. **(B)** JIMT-1 expressing vector control or Cx43 were injected into the mammary fat pad of immunocompromised mice to assess mammary tumor xenograft formation and growth. Tumor volume analysis on day 32 post-injection is presented. Student’s T-test was performed to determine p-values, p= or < 0.01 as indicated, n=10 animals per group. **(C)** SK-BR-3 cells expressing vector control, Cx43, or Cx43 G60S were assessed by transwell assay to evaluate migration. Representative images show that Cx43 expressing cells migrate to a lesser extent than control or Cx43 G60S cells. Number of cells migrated was quantitated and students T-test was used to determine p-values, p<0.0001 as indicated, n= 4 samples per experiment. **(D)** JIMT-1 cells expressing vector control, Cx43, or Cx43 G60S were assessed by transwell assay to evaluate migration. Representative images show that Cx43 and Cx43 G60S expressing cells migrate to a greater extent than control cells. Number of cells migrated was quantitated and students T-test was used to determine p-values, p<0.0001 as indicated, n= 4 samples per experiment.

Based on our findings thus far, we predicted that overexpression of Cx43 in JIMT-1 cells, which remain GJIC inactive, would lead to an increase in breast cancer properties including mammosphere formation and tumor growth. When we tested each of these properties, we found that SK-BR-3 cells had a poor capacity for forming mammospheres, similar to previous reports [33], and that Cx43 expression did not alter the capacity for mammosphere formation in the SK-BR-3 cell line (Figure 3C). Interestingly, the Cx43 G60S mutant increased mammosphere formation in the SK-Br-3 cells (Figure 3C). The differential activity of the G60S mutation in the SK-BR-3 cells suggested to us that the GJIC-deficient Cx43 could have non-junctional functions that contribute to the mammosphere forming capacity. When we evaluated tumor formation in the SK-BR-3 control, Cx43, and Cx43 G60S cells using a mammary tumor xenograft assay, the Cx43 expressing SK-BR-3 cells had a reduced capacity for tumor growth compared to vector control cells and Cx43 G60S cells (Figure 4A). Conversely, Cx43 overexpression in JIMT-1 cells significantly increased primary mammosphere formation (∼3-fold increase, p<0.001, Figure 3D), as did the Cx43 G60S mutant (Figure 3D). Furthermore, Cx43 and Cx43 G60S promoted tumor growth (∼2-fold increase in volume, Figure 4B) over vector control expressing JIMT-1 cells. Taken together, these findings indicate that despite Cx43 expression, dysregulation of Cx43 promotes tumorigenic properties in JIMT-1 cells, allowing the resulting breast cancers to be more aggressive.

### Non-junctional Cx43 is a feature of HER2 inhibitor resistant breast cancer cells that promotes angiogenesis and migration

Prior reports suggest a potential role for Cx43 in regulating breast cancer cell migration and consequently, metastasis [8-15, 19, 34, 35]. Further reports also suggest a possible role for Cx43 in angiogenesis [36–38]. Therefore, we evaluated these properties in our vector control and Cx43 expressing SK-BR-3 and JIMT-1 breast cancer cell lines. To evaluate angiogenesis, we performed an endothelial tube assay to assess how conditioned medium from vector control and Cx43 expressing SK-BR-3 or JIMT-1 cells would impact tube formation. We found that the conditioned medium from JIMT-1 Cx3 cell population was able to promote tube formation more robustly than the vector control JIMT-1 cells as well as both the vector and Cx43 expressing SK-BR-3 cells (Supplementary Figure 4).

We next evaluated the migration capacity of each breast cancer cell line by transwell migration assay. Interestingly, the SK-BR-3 cells expressing Cx43 migrated less efficiently than the vector control SK-BR-3, whereas the Cx43 G60S GJIC-deficient cells did not suppress migration (Figure 4C). However, the JIMT-1 cells expressing Cx43 migrated more efficiently than the vector control JIMT-1 cells, as did the Cx43 G60S expressing JIMT-1 cells (Figure 4D). These findings suggest that maintaining Cx43 GJIC inhibits cell migration. Furthermore, as evidenced by results in the JIMT-1 cells, a non-gap junctional Cx43 can promote metastatic features including angiogenesis and cell migration.

## DISCUSSION

We have investigated differences in Cx43 regulation in HER2+ breast cancer cells that are drug sensitive and drug resistant to HER2 inhibitors. Based on our observations, we conclude that when experimental methods are used to overexpress Cx43, whether the breast cancer cell is sensitive to HER2 inhibitors or resistant to HER2 inhibitors dictates if the exogenously expressed Cx43 can induce the formation of functional gap junctions. Based on the findings reported here, we suggest a progressive model whereby HER2+ breast cancer cells can gradually accumulate mechanisms to disable Cx43 GJIC as a feature of drug resistance. We surmise that as the cell becomes transformed, Cx43 expression is reduced, presumably in order to reduce GJIC. However, the cell will likely remain gap junction competent despite the reduced Cx43 protein levels. At early stages of malignancy, it seems likely that cancer cells will retain their ability to communicate through gap junctions, if a signal such as Cx43 overexpression, is provided to facilitate the formation of gap junctions. However, as the cancer cell develops resistance to chemotherapeutic agents, additional mechanisms arise in order to completely impair GJIC, such as mislocalization of Cx43. Since Cx43 protein is directed elsewhere in the cell, non-canonical functions of Cx43 likely predominate, leading to changes in proliferation and survival that can potentially influence tumor aggressiveness. Further studies to understand the mechanisms that impair gap junction formation in drug resistant breast cancer cells are required in order to determine if it is possible to force the reestablishment of gap junctions in drug resistant cells by methods beyond Cx43 overexpression. Some prior studies have implicated that re-establishment of Cx43 GJIC is required for metastasis [4, 16, 18, 19, 34, 39]. Therefore, great care must be taken to study the activity of Cx43 in accordance with the different stages of metastasis (i.e. invasion, intravasation, extravasation, and colonization). Likely, extensive studies to evaluate the Cx43 lifecycle and how this protein is trafficked in models of specific breast cancer subtypes will be required to determine the mechanism by which cells retain Cx43 away from gap junctions.

Importantly, these studies highlight a less well studied area of Cx43, namely, non-canonical functions for Cx43 that are independent of its gap junctional activity. The C-terminal portion of Cx43, which is located in the cytoplasm, contributes to many non-canonical Cx43 functions [20]. This portion of Cx43 represents the main site of interaction with intracellular proteins and has been reported to contribute to cell proliferation (cell cycle), cell death, migration, and transcription [20, 32, 40–42]. The general overall observation from these studies is that the C-terminal domain of Cx43 impairs cell proliferation but promotes cell migration [20]. Our findings support observations that gap junction independent Cx43 affects these cellular processes. However, we find that Cx43 expression in JIMT-1 cells increased the proliferative and tumor growth capacity as well as induced migration of these cells. The former is contradictory to the general trend observed in prior studies on the C-terminal domain. Consequently, we must consider whether the C-terminal domain of Cx43 in JIMT-1 cells is being differentially modified and how this relates to our findings. Albeit we only used a full length protein for our studies but a GJIC-deficient G60S mutant of Cx43 confirmed many of our observations. Since it is largely unknown whether the G60S point mutation alters regulation of the C-terminal domain of Cx43, additional studies are required to evaluate whether regulatory events including protein-protein interactions are altered. It will be interesting to determine where Cx43 is acting in the JIMT-1 cells to potentiate these non-canonical functions, what signaling pathways are regulating these functions, and possibly how Cx43 is differentially processed in these cells. Recent efforts have focused on connexin hemichannel function but non-membrane related localization is also reported for Cx43 and thus, we must consider all avenues of function for this protein [7, 20].

It is worth noting that distinct cell properties were affected in the SK-BR-3 cells by restoring GJIC through exogenous Cx43 expression. We observed a clear effect on migration in the SK-BR-3 cells. Re-establishing GJIC via Cx43 expression reduced cell migration and the GJIC-deficient G60S mutant of Cx43 did not. This finding is consistent with reports that Cx43 GJIC inhibits cell migration [8–15]. Similarly, an increase in migration was observed in JIMT-1 cells that overexpress Cx43 but lack GJIC. The latter observation does not clarify whether it is the impaired GJIC or non-junctional roles for Cx43 that promote the cell migration in JIMT-1 cells. These findings further highlight some of the confusion in the Cx43 breast cancer field, mainly lack of consensus for Cx43’s role in metastasis. However, it needs to be noted that metastasis is a complicated process that not only involves the migration of the tumor cell but how the tumor cell interacts with its environment, leaving room for many potential roles that Cx43 and GJIC could play during the metastatic process.

Certainly, we must discuss the merits and faults of using cultured cell lines as well as the positives and negatives of using overexpression as an experimental model system when studying Cx43. While both the SK-BR-3 cell line and the JIMT-1 cell line are classified as HER2+, there are distinct differences in their characteristics and culturing methods. The SK-BR-3 line was derived in 1970 by pleural effusion from a 43 year old female with metastatic breast cancer that had been treated with standard chemotherapy whereas the JIMT-1 line was derived by pleural effusion from a 62 year old female with grade 3 invasive breast cancer that had received trastuzumab and developed resistance to trastuzumab [43, 44]. While both cell lines were derived from aggressive HER2+ breast cancers, the treatment protocols each patient received likely contributed to the overall behavior of the resultant cell lines in culture beyond the obvious sensitivity or resistance to HER2 inhibitors. Exogenous expression of proteins is clearly a contrived experimental system. However, overexpression can be used to exacerbate phenotypes driven by the protein of interest, in our case Cx43. Moving forward, identifying reagents to modify endogenous Cx43 protein may be critical. We and others have performed studies in cancer models using agents such a therapeutic peptides that mimic or antagonize endogenous Cx43 function to study this protein [7, 45, 46]. It is predicted that the ability to evaluate and test protein function for Cx43 will become more efficient and effective as we increase our knowledge about how this protein is regulated in different cell types and disease states.

## MATERIALS AND METHODS

### Cell culture

All cells were grown at 37°C and 5% CO_2_. SK-BR-3 cells were obtained from American Type Culture Collection (ATCC^®^, Manassas, VA) and cultured in DMEM (Corning 10-017-CV) supplemented with 10% FBS (Gibco/Thermo-Fisher Scientific), 5 μg/ml Insulin (Gemini Bio-Products, West Sacromento, CA), 2mM Glutamine (Corning), and Penicillin/Streptomycin (Pen/Strep, Thermo Scientific). JIMT-1 cells were obtained from Addex Bio (Addex Bio C0006005, San Diego, CA) and cultured in DMEM (Corning 10-017-CV) supplemented with 10% FBS (Gibco/Thermo-Fisher Scientific), 2mM Glutamine (Corning), and Penicillin/Streptomycin (Pen/Strep, Thermo Scientific). JIMT-1 cells were cultured for less than 6 months since purchase and tested negative for bacteria, mycoplasma, yeast, HIV, Hepatitis B and Hepatitis C (Addex Bio C000605). SVR angiosarcoma cells were obtained from American Type Culture Collection (ATCC^®^, Manassas, VA) and cultured in Opti-MEM 8% fetal bovine serum (FBS) (Omega Scientific, Tarzana, CA). In addition, SVR angiosarcoma cells were tested negative by Research Analytic Diagnostic Laboratory (Columbia, MO) by PCR evaluation for: Ectromelia, EDIM, LCMV, LDEV, MHV, MNV, MPV, MVM, *Mycoplasma* sp., Polyoma, PVM, REO3, Sendai, TMEV GDVII.

### RNA isolation and real time PCR

RNA was prepared by using the GeneJet RNA isolation kit (Thermo-Fisher Scientific). Reverse transcription was performed using iScript Reverse Transcriptase Supermix (Bio-Rad). The resulting cDNA was used to perform quantitative RealTime PCR using the Bio-Rad myIQ system. PrimePCR SYBR Green Assay for human GJA1 (qHsaCID0012977) was purchased from Bio-Rad. Primers for GAPDH are Forward-TGCACCACCAACTGCTTAGC and Reverse-GGCATGGACTGTGGTCATGAG.

### Immunoblotting

Cells were lysed in 2X Laemmli sample buffer followed by sonication (Artek Systems, BioLogics Inc., Manassas, VA) at 30% amplitude for 10 sec. Primary antibodies used for western blotting are: anti-Cx43 (Sigma-Aldrich C6219) and anti-β-tubulin (Santa Cruz sc-55529). Imaging and quantitation was performed on the FluorChem-R instrument (ProteinSimple, San Jose, CA). Quantitation of protein expression was performed using AlphaView software. Cx43 was normalized to β-tubulin.

### Immunofluorescense

Cells were plated on No. 1.5 square 22x22 mm coverslips (Corning). Primary antibodies used for immunofluorescence are: anti-Cx43 (Sigma-Aldrich C6219) and anti-EGFR (Santa Cruz sc-373746). Secondary antibodies are Alexa Fluor 488 (Thermo-Fisher Scientific) and Alexa Fluor 594 (Thermo-Fisher Scientific). Imaging was performed using 63X oil immersion objective (total magnification 630X) on a Leica TCS SPE confocal microscope and processed using the LAS X software platform (Leica Microsystems Inc., Buffalo Grove, IL).

### Coupling assays

20,000 cells per well were plated into 96 well plates. A separate dish of Cx43 expressing cells, for each representative cell type, either SK-BR-3 or JIMT-1, was loaded with 1 ng/μl calcein-AM (BD Biosciences, Bedford, MA) for 30 min. The calcein-AM loaded cells were washed, trypsinized, and counted. 5000 dye-loaded cells/well were dropped onto the cells plated in the 96 well dish. 6 hrs later, cells were counted and analyzed for calcein-AM fluorescence using a Luna-FL (Logos Biosystems, Annandale, VA) cell counter. For each cell type n=6 replicates were evaluated per experiment and each experiment was performed 3 times. Fold change represents the number of calcein-AM positive cells above the original 5000 dye-loaded cells dropped per well.

### Proliferation and cell counting assays

5,000 cells per well were plated into 96 well plates. At the indicated time points, cells were treated with MTT reagent and absorbance read at 570 nM using a Filtermax F5 plate reader (Molecular Devices, Sunnyvale, CA). For cell counting assays, 100,000 cells per well were plated into 24 well plates. The following day, cells were either counted (time=0 hrs) or serum deprived by washing and replacing medium with serum free medium. 48 hrs later, cells in serum free medium were counted and cell numbers were analyzed for fold change compared to time=0 hrs samples. Cell counting was performed using a Luna-FL (Logos Biosystems, Annandale, VA) cell counter. For all assays, MTT and cell counting, n=6 replicates were evaluated for each cell type per experiment and each experiment was performed 3 times.

### Mammosphere assay

Mammosphere assay was performed as previously reported [39]. Briefly, 500 cells per well were plated into cell repellent 96 well plates (Greiner Bio-One, Monroe, NC). On day 10 of the experiment, mammosphere structures were quantitated. For each cell type, n=32 replicates were evaluated per experiment and each experiment was performed 3 times.

### Angiosarcoma tube formation assay

ECMatrix (Millipore Corp, Billerica, MA) was thawed, diluted, and solidified into wells of a 96-well plate according to the manufacturer’s instructions. SVR angiosarcoma cells were serum starved (2.5% FBS) overnight and seeded onto the matrix at a concentration of 35,000-40,000 cells per well (n=9) in 150μL fresh DMEM with 10% FBS from four different conditions: SK-BR-3 -vector control, SK-BR-3-Cx43, JIMT-1-vector control, JIMT-1-Cx43. Once plated, the cells were incubated at 37°C, 5% CO2 for 4 hours. Images were acquired using 4.0x objective lens of EVOS FLc microscope (Life Technologies, Carlsbad, CA). Results were quantified by counting the number of branch points using Image J Angiogenesis Analyzer software (National Institutes of Health, Bethesda, MD, USA).

### Migration assays

Transwell migration assays were performed as previously described [47]. Briefly, 50,000 cells were plated into upper chamber portion of transwell dish in serum free medium. Complete medium containing 10% FBS was placed in the bottom chamber portion of the transwell dish. Cells were incubated for 24 hrs before membranes were stained, imaged, and quantitated. Images were captured on a Labomed light microscope using a 20X objective (total magnification 200X).

### Animal care and xenograft tumor experiments

Animal care and all animal experiments were performed with the approval and in accordance with the guidelines of the Medical University of South Carolina IACUC. All mice were housed and cared for in the Animal Research Center at Medical University of South Carolina, which is AAALAC accredited facility. Mice were housed in a BSL2 rooming facility for immunocompromised animals. Animals were euthanized by anesthesia overdose with isofluorane in accordance with the *Guide for the Care and Use of Laboratory Animals*. Protocols were in place for early and humane endpoints in the event that an experimental animal displayed signs of illness, such as poor body condition, lethargy, piloerection, and lack of grooming behavior, prior to the experimental endpoint. To determine when/if animals should be euthanized, tumor measurements and health monitoring of experimental animals was performed regularly by lab and veterinary staff. For orthotopic tumor analysis, 5 x 10^6^ cells were injected in the abdominal mammary fat pat of immunocompromised mice (Nu/J-Foxn1^nu/nu^ or NSG, Jackson Labs). Tumors were evaluated by manual palpation using calipers.

### Statistics

For all statistical analyses, p-values for *in vitro* experiments were analyzed using Student’s T-test. p<0.05 was considered statistically significant. As indicated previously in the methods, all experiments were performed in triplicate with multiple replicates (typically n>4) for each experiment.

## SUPPLEMENTARY MATERIALS FIGURES


