# Supplementary material for: Dysregulated connexin 43 in HER2-positive drug resistant breast cancer cells enhances proliferation and migration
Source: Oncotarget. 2017 Nov 25;8(65):109358–69. doi: 10.18632/oncotarget.22678 (PMC5752526; doi:10.18632/oncotarget.22678)
Supplement: Supplementary file 1 [file oncotarget-08-109358-s001.pdf]

Dysregulated connexin 43 in HER2-positive drug resistant breast cancer cells enhances proliferation and migration

SUPPLEMENTARY MATERIALS

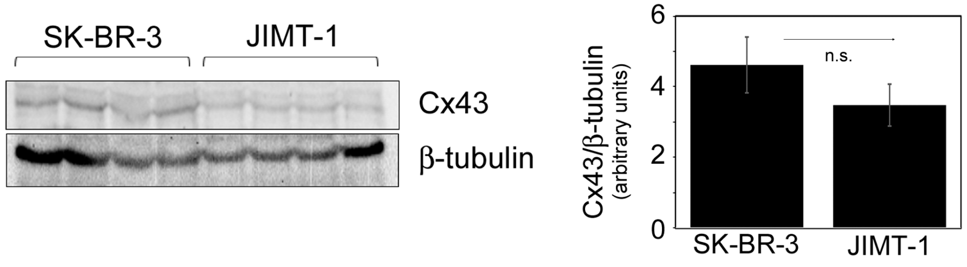

Supplementary Figure 1: Quantitation of endogenous Cx43 protein levels in SK-BR-3 and JIMT-1 cells.

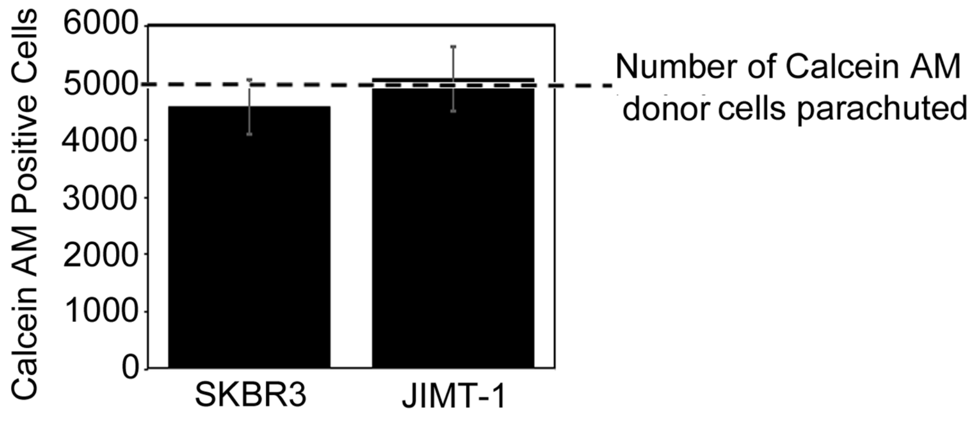

Supplementary Figure 2: Baseline coupling in SK-BR-3 and JIMT-1 cells.

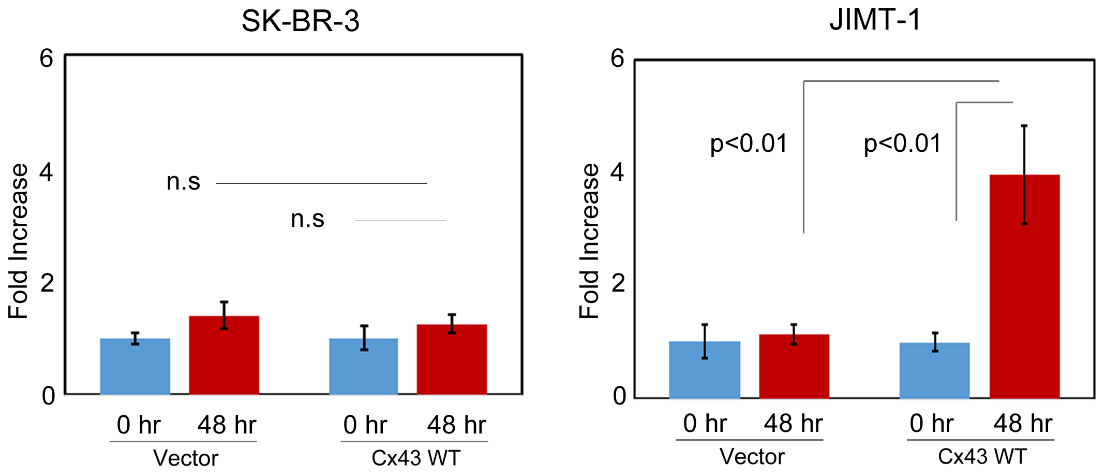

Supplementary Figure 3: Cell viability under serum limiting conditions of SK-BR-3 and JIMT-1 cells expressing a vector control or Cx43.

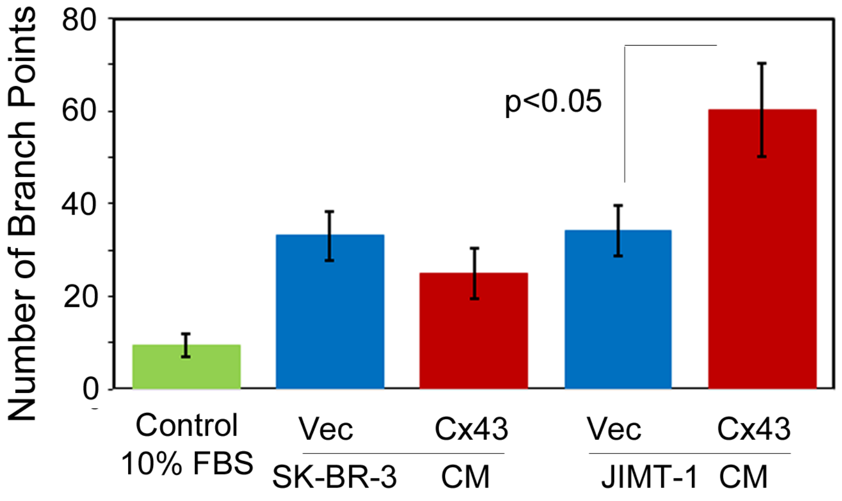

Supplementary Figure 4: Angiogenesis is induced by Cx43 in JIMT-1 cells.
